# Prof. Huxley on the Anti-Vivisectionists

**Published:** 1877-10

**Authors:** 


### Professor Huxley on the Anti-Vivisectionists.

In a recent address relating to the law prohibiting vivisection for scientific purposes, lately passed in England, Professor Huxley summed up the whole contradictoriness of the situation as it had all along been seen by liberal and sensible men. "I think it my duty," says the Professor, "to express my regret at a condition of the law which permits a boy to troll for pike, or set lines with live frog bait for amusement; and, at the same time, lays the teacher of that boy open to the penalty of fine and imprisonment if he uses the same animal for the purpose of exhibiting one of the most beautiful and instructive of physiological spectacles, the circulation in the web of the foot. The first offender says, 'I did it because I find fishing very amusing,' and the magistrate bids him depart in peace. The second pleads, 'I want to impress a scientific truth, with a distinctness attainable in no other way, on the minds of my scholars,' and the magistrate fines him £5." The illustration is a homely one, but it exactly expresses the facts in the case, and it seems hardly possible that the anomaly will be permitted to much longer stand in British law. If it should so remain, our English cousins must be content to be open to the charge of neglecting important means of education on a very insufficient plea.—*Medical and Surgical Reporter.*

